# The circle of reentry: Characteristics of trigger-substrate interaction leading to sudden cardiac arrest

**DOI:** 10.3389/fcvm.2023.1121517

**Published:** 2023-04-17

**Authors:** Matthijs J. M. Cluitmans, Jason Bayer, Laura R. Bear, Rachel M. A. ter Bekke, Jordi Heijman, Ruben Coronel, Paul G. A. Volders

**Affiliations:** ^1^ Cardiovascular Research Institute Maastricht, Maastricht University, Maastricht, Netherlands; ^2^ Philips Research, Eindhoven, Netherlands; ^3^ IHU LIRYC, Pessac, France; ^4^ Department of Experimental Cardiology, Amsterdam University Medical Centre, Amsterdam, Netherlands

**Keywords:** arrhythmia, trigger, substrate, sudden cardiac arrest, electrocardiogaphy

## Abstract

Sudden cardiac death is often caused by ventricular arrhythmias driven by reentry. Comprehensive characterization of the potential triggers and substrate in survivors of sudden cardiac arrest has provided insights into the trigger-substrate interaction leading to reentry. Previously, a “Triangle of Arrhythmogenesis”, reflecting interactions between substrate, trigger and modulating factors, has been proposed to reason about arrhythmia initiation. Here, we expand upon this concept by separating the trigger and substrate characteristics in their spatial and temporal components. This yields four key elements that are required for the initiation of reentry: local dispersion of excitability (e.g., the presence of steep repolarization time gradients), a critical relative size of the region of excitability and the region of inexcitability (e.g., a sufficiently large region with early repolarization), a trigger that originates at a time when some tissue is excitable and other tissue is inexcitable (e.g., an early premature complex), and which occurs from an excitable region (e.g., from a region with early repolarization). We discuss how these findings yield a new mechanistic framework for reasoning about reentry initiation, the “Circle of Reentry.” In a patient case of unexplained ventricular fibrillation, we then illustrate how a comprehensive clinical investigation of these trigger-substrate characteristics may help to understand the associated arrhythmia mechanism. We will also discuss how this reentry initiation concept may help to identify patients at risk, and how similar reasoning may apply to other reentrant arrhythmias.

## 1. Introduction

Sudden cardiac death is often caused by ventricular arrhythmias driven by reentry. In a recent study in survivors of sudden cardiac arrest (SCA), explanted human and pig hearts, and computer simulations we uncovered key elements required for the initiation of reentry leading to ventricular tachycardia (VT) and fibrillation (VF) in the absence of pronounced structural remodeling (1). This has highlighted the complex spatiotemporal interaction between trigger and substrate, where the vulnerable substrate can be formed by cardiac regions with pronounced differences in local time of re-excitability, and the trigger can be formed by a premature activation; the trigger's relative origin and timing can then interact with the substrate, leading to unidirectional block and reentry. In this “Hypothesis and Theory” article, we extend previous hypotheses on trigger-substrate interaction to incorporate these findings, arriving at a “Circle of Reentry” concept. In addition, we will present a patient case to illustrate this arrhythmia mechanism and discuss the generalization of the concept to other arrhythmias. We will also use the patient case to show how an in-depth trigger-substrate characterization (by employing noninvasive mapping of subtle electrical substrate and personalized computational modeling) may help to identify patients at risk for VT/VF, and how it might guide therapeutic decisions.

## 2. The circle of reentry

**Figure 1:**
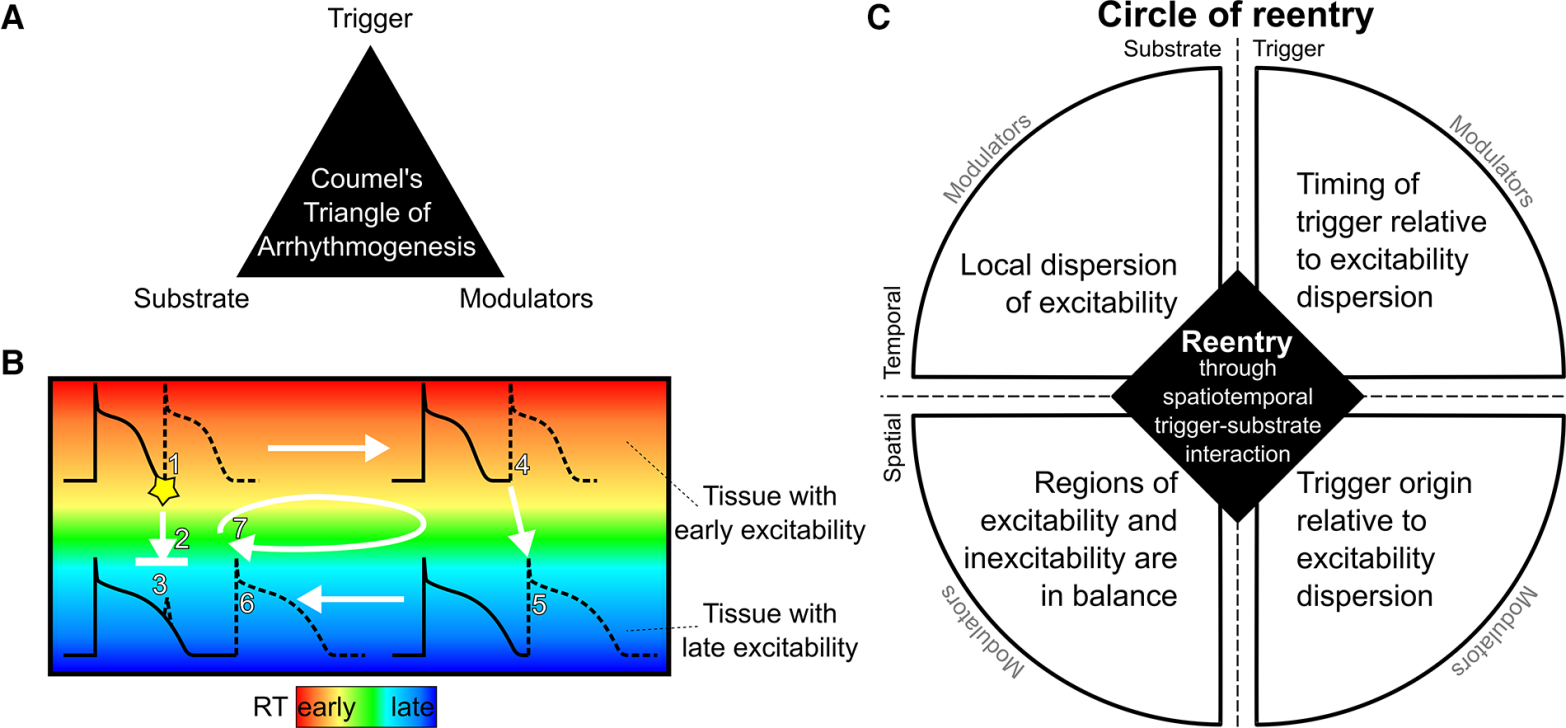
The circle of reentry hypothesis. (**A**): The Triangle of Arrhythmogenesis by Coumel identifies three major factors for arrhythmia initiation. (**B**): Schematic illustration of reentry in tissue with early and late repolarization time (RT) due to short or long action potential duration, respectively. A premature beat coming from an early RT region (1) may block against tissue that has a longer RT and is still refractory (2) and cannot induce activation in the late RT region (3). If the early RT region is large enough, the activation wave may propagate through that region (4) in a time span that allows the late RT region to repolarize and become excitable, allowing re-activation of the tissue (5). The wave may then travel back to the region that was still refractory but which has become excitable in the meantime (6). By that moment, the tissue from which the premature beat originated has become excitable again, and the wavefront from the late RT region will propagate to that tissue, restarting this circuit (7). (**C**): The Circle of Reentry proposes four requirements for reentry arising from spatiotemporal interactions between trigger and substrate: (1) local dispersion of excitability (e.g., steep RT gradients), (2) a balance in size of the region of excitability and the region of inexcitability (e.g., sufficiently large region of early RT), (3) a trigger originating at a time when some tissue is excitable and other tissue is inexcitable (e.g., an early premature beat), and (4) which occurs from an excitable region (e.g., from early RT region). Each of the four elements can be affected by modulators.

In the 1980s, Coumel (2) introduced the “Triangle of Arrhythmogenesis” (Figure 1A), which captures the trigger-substrate-modulator interaction leading to arrhythmias: “there are always three main ingredients required for the production of a clinical arrhythmia: the arrhythmogenic substrate, the trigger factor and the modulation factors.” (3) This concept is applicable to many forms of arrhythmogenesis. For example, triggers may originate from the pulmonary veins [in atrial fibrillation (4)] or Purkinje fibers [in VF (5)], and may interact with fibrotic substrate [e.g., in atrial fibrillation or post-infarct hearts (6)] or fatty infiltration [e.g., around scar (7)]. All these factors are also affected by modulators, including the autonomic nervous system (e.g., stress), intra- or extracellular homeostasis (e.g., electrolyte disbalance, inflammation), dietary factors, drugs, pressure, or myocardial stretch. Here, we extend the “Triangle of Arrhythmogenesis” concept by subdividing the characteristics of both trigger and substrate in their respective spatial and temporal factors, showing their relevance for the occurrence of reentry.

Our recent translational study introduced the concept of four key interacting elements that predispose the ventricular myocardium to reentry that leads to VT and VF, based on a purely electrical substrate of recovery abnormalities (1). Regional abnormalities in electrical recovery (i.e., repolarization) of the heart can indeed form an important excitability substrate for cardiac arrhythmias (8). Our recent study of idiopathic VF survivors (who had SCA but no detectable structural or other abnormalities in their clinical workup) uncovered steep repolarization time (RT) gradients that could not be detected on the 12-lead ECG (1). We showed that these RT gradients were significantly steeper in idiopathic VF survivors than in control individuals. These gradients arose at the border between regions of relatively early RT and regions of relatively late RT. The sizes of the tissue with early and late repolarization were approximately equal. Moreover, the macroscopic origin of spontaneous premature complexes typically was identified in these early RT regions, and such premature beats were earlier in survivors of idiopathic VF than in the control group. Ex vivo and in silico experiments then uncovered the underlying arrhythmia mechanism, which is illustrated in Figure 1B: an early premature beat occurring from early-recovered (early RT) tissue may block against the gradient bordering a late-RT region that is still refractory. If the early-RT region is large enough the premature beat may take sufficient time to travel around the late-RT region for it to become excitable and may subsequently re-activate the early-RT region again, leading to reentry. Thus, we identified critical spatial and temporal characteristics of the substrate and trigger that predispose to reentry and VT/VF in ventricles without structural abnormalities (Table 1).

**Table 1:** The spatiotemporal characteristics of trigger-substrate interaction leading to VF in hearts with repolarization abnormalities (but no structural abnormalities), as identified in (1). RT, repolarization time.

	Substrate	Trigger
Temporal characteristic	Region of early RT next to region of late RT, separated by steep RT gradients	Trigger occurs early (short-coupled)
Spatial characteristic	Size of early-RT vs. late-RT region is sufficiently large	Trigger macroscopically originates from region of early RT

We may extend these findings by considering that dispersion of repolarization is more generally captured by dispersion of *excitability* as the underlying determinant of reentry. Excitability, or lack thereof (refractoriness), can be influenced by shortened or prolonged action potential duration, but also by delayed activation (e.g., in or around scar) or post-repolarization refractoriness (e.g., as in ischemia). This then leads to the generalized concept of a “Circle of Reentry,” a mechanistic framework that explains the critical interaction of four factors that predispose the ventricular myocardium to reentry (Figure 1C): (1) the timing of the inducing trigger relative to the dispersion of excitability, (2) its macroscopic origin, coming from a region of early excitability, (3) the presence of a steep excitability time gradient, and (4) the relative sizes of the tissue with early and late excitability.

We will illustrate this concept in a patient who suffered SCA due to idiopathic VF. To study the trigger-substrate interaction at the organ level in this patient, trigger origins and local repolarization abnormalities were imaged with noninvasive electrocardiographic imaging and further studied with computational modeling. This personalized modeling provides a better understanding of the general arrhythmia concepts and the modulating factors operative within an individual patient, such as variations in repolarizations abnormalities and trigger origins and timings. We will end this “Hypothesis and Theory” paper by discussing how our conceptual framework may apply to reentry initiation in other conditions.

## 3. Illustration with a patient case of idiopathic VF

**Figure 2:**
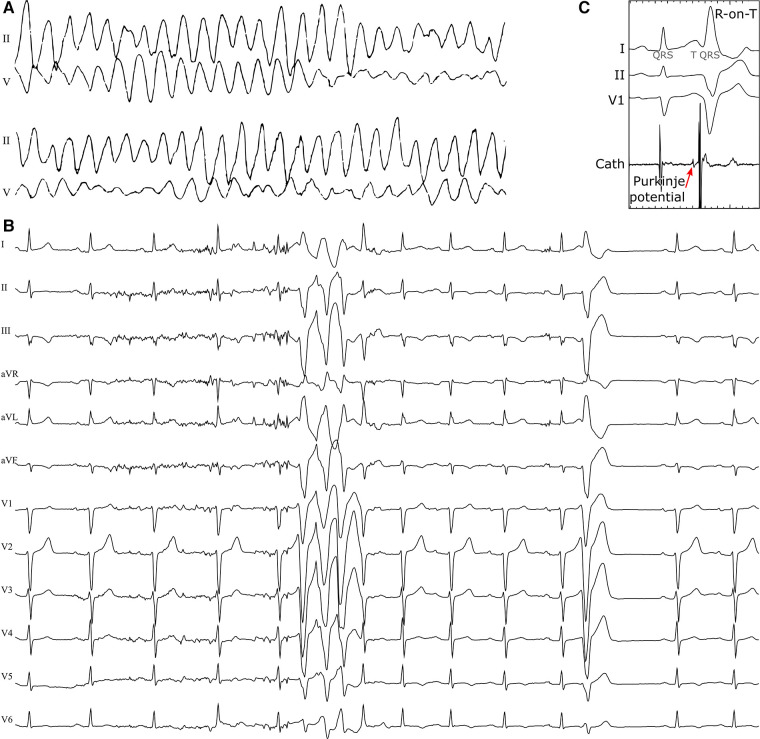
Polymorphic ventricular tachycardia at presentation (**A**), short-coupled extrasystoles from the right-ventricular moderator-band region with an R-on-T morphology and runs of NSVT (**B**) in a 47-year old female patient with no previous cardiac history. An invasive electrophysiology study highlighted Purkinje-fiber activity preceding the R-on-T premature beats (**C**).

A 47-year old woman, without previous history of cardiovascular disease, was transferred to the Maastricht University Medical Center because of out-of-hospital syncopes and in-hospital cardiac arrest due to polymorphic VT and VF, see Figure 2A. After defibrillation, she exhibited sinus rhythm with frequent, short-coupled premature ventricular complexes (PVCs) and short runs of non-sustained VT (NSVT, Figure 2B). Her coronary angiogram showed no abnormalities and plasma electrolyte concentrations were normal. No structural abnormalities were detected with cardiac ultrasound and magnetic resonance imaging (MRI) and ultrasound. A 24-hours Holter recording showed 17,675 mostly unifocal PVCs with short coupling interval. Ajmaline provocation testing was negative for Brugada syndrome, and completely abolished PVCs. A subsequent electrophysiological study confirmed the RV moderator-band origin of PVCs with preceding Purkinje potentials (suggestive for a Purkinje fiber origin, Figure 2C), and showed normal endocardial RV voltage maps. Thus, clinical work-up and conventional imaging did not reveal a substrate and this case was classified as idiopathic VF (9). DNA screening was performed to determine potential genetic factors involved in the arrhythmogenic phenotype. The clinical study was approved by the local ethical committee and registered as ClinicalTrials.gov NCT03947021.

## 4. Noninvasive ECGI for characterization of substrate and trigger

**Figure 3:**
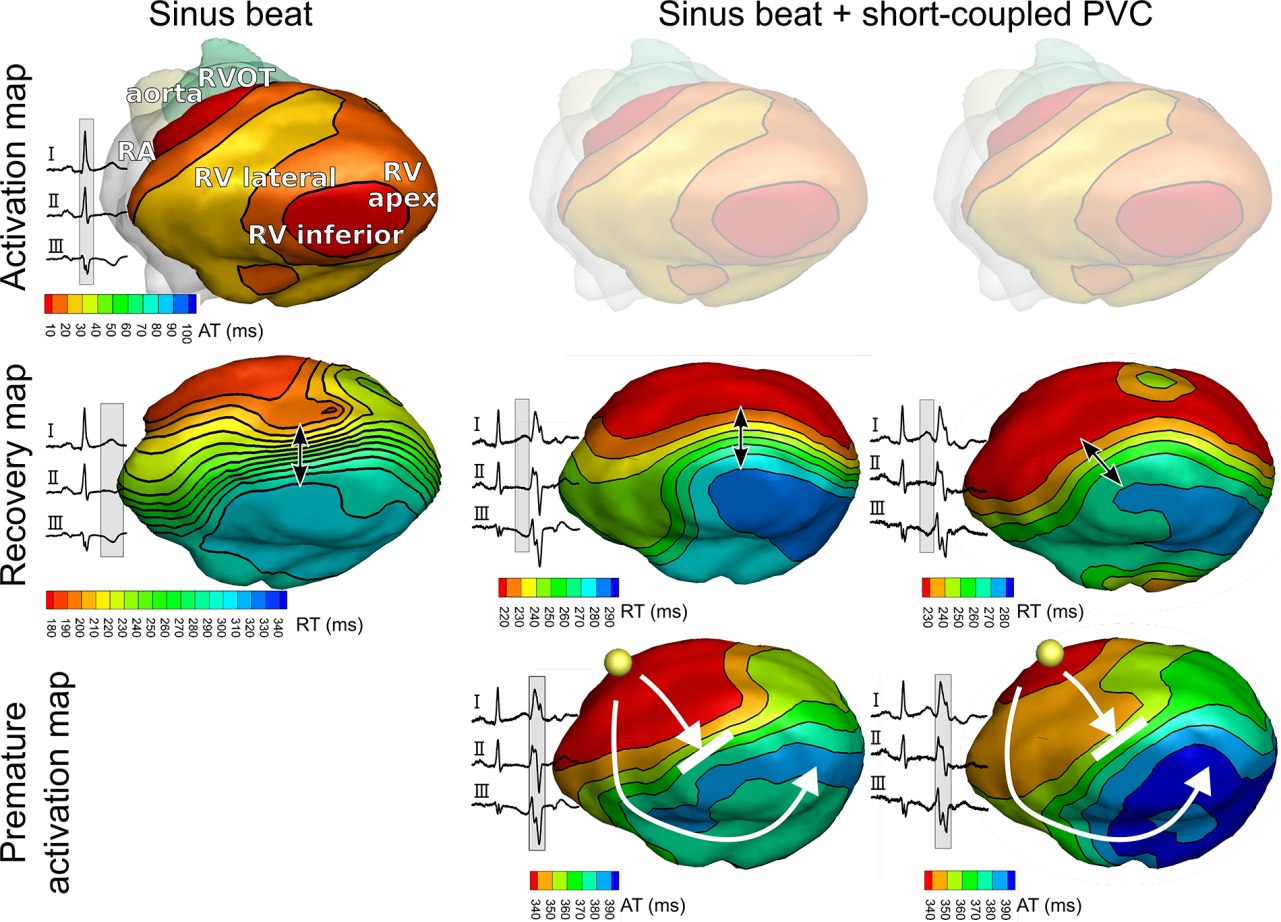
Noninvasively reconstructed epicardial isochrone maps for activation and recovery of sinus beats and premature ventricular complexes (PVCs). Epicardial isochrones show normal ventricular activation during sinus rhythm (row 1), but abnormal recovery (row 2) with steep repolarization time gradients (arrow). PVC activation (row 3) follows the pattern of recovery (row 2) of the preceding sinus beat. Yellow spheres in row 3 indicate the origin of PVC.

Neighboring tissue regions with locally different repolarization durations can provide a substrate for reentrant tachycardia, but may not significantly affect global 12-lead ECG metrics such as QT(c) intervals, making them impossible to detect with routine clinical examination. Noninvasive electrocardiographic imaging (ECGI) enables reconstruction of potentials, electrograms, and activation/recovery isochrones on the epicardium (10). It can overcome the low spatial resolution of the 12-lead ECG by reconstructing electrograms directly at the epicardium from a much larger set of (up to 256) body-surface ECGs combined with computed tomography (CT)-derived information about the heart and body-surface geometries. In this patient, ECGI (Figure 3) showed normal ventricular activation during sinus rhythm (row 1), with first epicardial breakthroughs at the RV outflow tract (RVOT) and RV apex. This activation pattern is consistent with normal patterns of activation as imaged previously with ECGI in healthy human adults (11) and in explanted human hearts (12). Repolarization of this sinus beat, on the other hand, was abnormal (row 2). In particular, the RVOT region recovered relatively early (180 ms from QRS onset) whereas the inferior RV recovered at 300 ms. In our recent study, none of the healthy subjects had a region with such an early repolarization time (1). In this patient with apparently idiopathic VF, there was a relatively steep repolarization gradient between these areas [185 ms/cm, close to the steepness cutoff of 200 ms/cm we recently found between healthy and idiopathic VF patients (1)], satisfying a first essential requirement of the Circle of Reentry.

Furthermore, ECGI identified that the short-coupled PVCs originate from the RVOT region. The PVC activation pattern (Figure 3, row 3) follows the pattern of recovery (row 2) of the preceding sinus beat. Activation-recovery intervals (not shown) are similarly distributed as RTs, indicating that repolarization gradients are induced by local changes in APD. ECG recordings showed that PVCs were short-coupled (290 ms).

**Figure 4:**
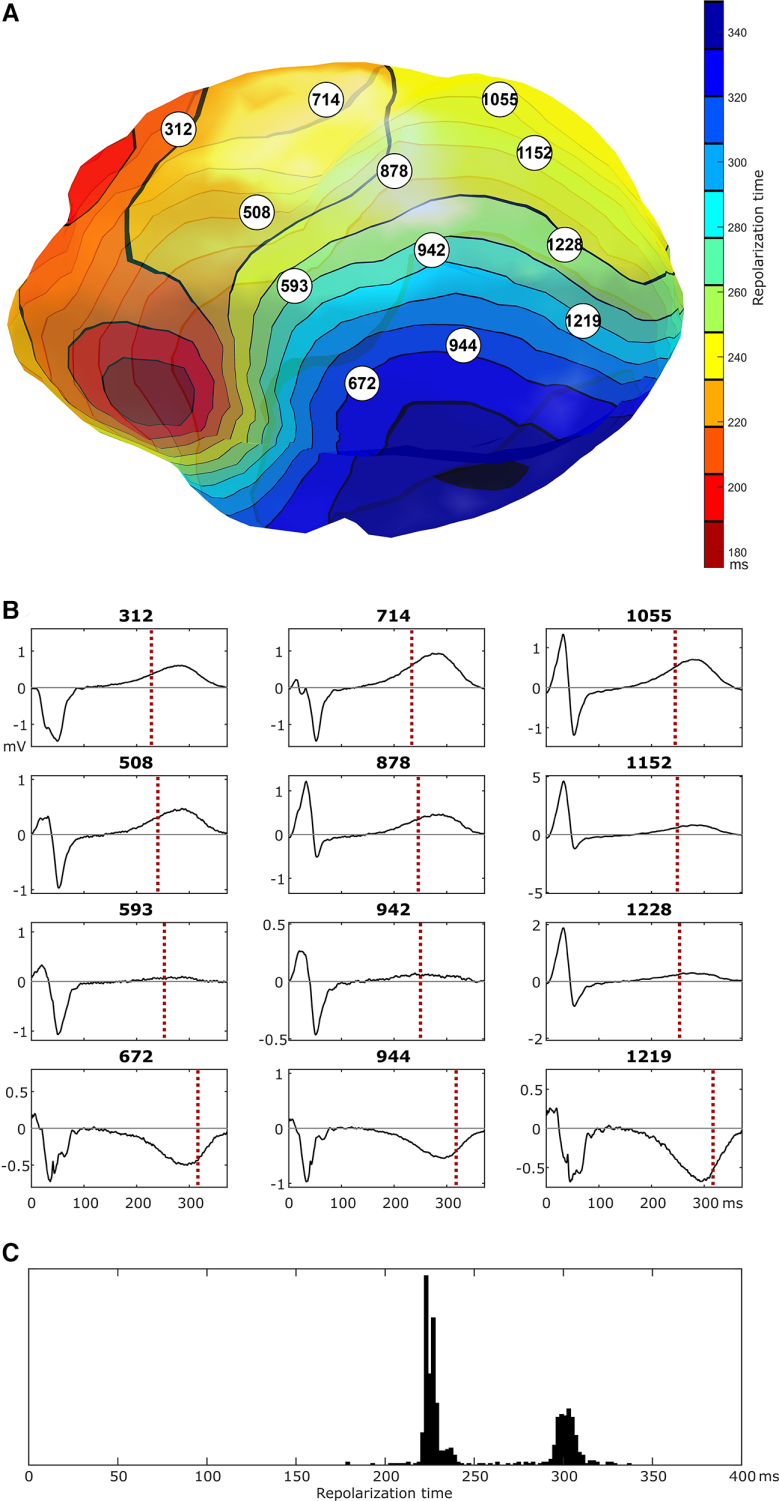
Epicardial isochrones of repolarization times [panel (**A**)] and selected epicardial electrograms [numbers reflect node id, panel (**B**)], as imaged with ECGI during a sinus beat. In the region of crowded repolarization isochrones, there is a switch in polarity of the repolarization phase of the local electrogram. Local electrograms show no clear signs of conduction abnormalities (the high-frequency signal in the last row of electrograms is present throughout the depolarization and repolarization phases and may reflect powerline interference). Panel (**C**) shows the distribution of repolarization times for all locations on the epicardial ventricular surface, highlighting a distinct region of early repolarization and a distinct region of late repolarization of approximately the same size.

Figure 4 shows selected epicardial electrograms mapped with ECGI during sinus rhythm. There are no signs of abnormal conduction, i.e., no fractionation or ST-segment elevation. The crowded isochrones are in a region where the local electrogram changes polarity during repolarization. Since local RT is reflected by the upslope of the unipolar electrogram (1, 13, 14), regions of polarity change reflect a change from early to late RT; if this happens over a small area, a steep RT gradient develops, as observed here. Additionally, the histogram (Figure 4C) reveals two distinct distributions of early and late RT. These regions are relatively balanced in size (with a surface ratio of the early region vs. late region of 0.59).

Although we were unable to record a VT episode in this patient during the ECGI procedure, we could noninvasively identify all four key elements of the Circle of Reentry (Figure 1C): the presence of excitability dispersion (based on a repolarization substrate), where the region of early repolarization and late repolarization are balanced in size, and early PVCs macroscopically originating from the early repolarizing region. Further analysis with personalized computational modeling was subsequently performed to shed further light on trigger-substrate interaction.

## 5. Personalized computational modeling: trigger-substrate interaction

**Figure 5:**
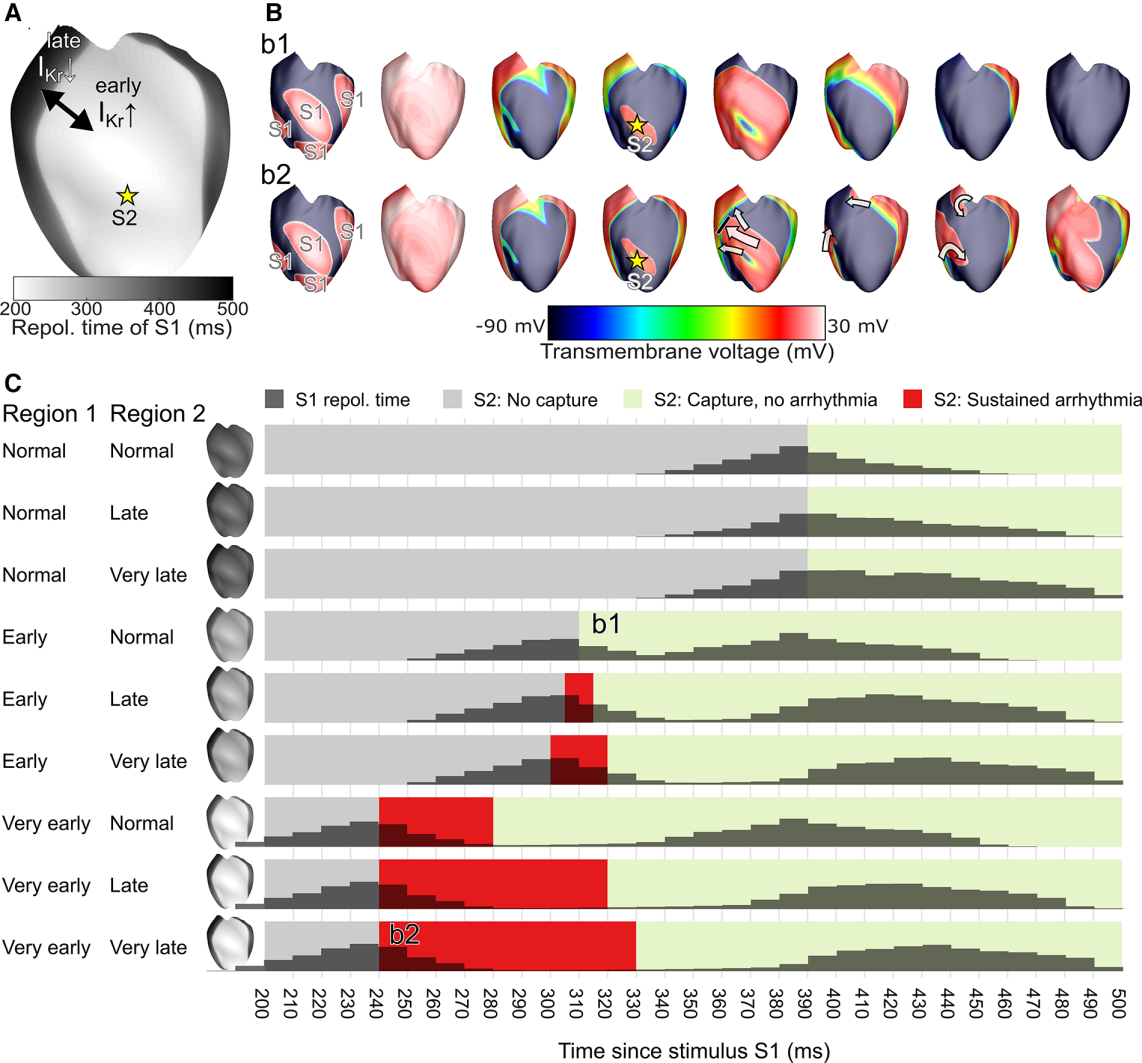
In a computational model of ventricular epicardium, a region of early and late repolarization was created by changing the conductance of the rapid delayed-rectifier potassium current (I_Kr_) in an early and late region (**A**). An S1-S2 stimulus protocol was used to test arrhythmia induction for different settings of the early RT region, the late RT region, and the S1-S2 coupling interval; snapshots of two examples are shown in (**B**). Simulation outcomes are displayed in panel (**C**) and show the S1 repolarization time (histograms), and the arrhythmia outcome for each tested 10** **ms S1-S2 interval tested, with the S1-S2 interval leading to sustained arrhythmias displayed in red. The example simulation snapshots of panel B are referenced in panel C as b1 and b2.

We replicated the ECGI observations of this patient in a previously published computational model of the ventricular epicardium (15). Simulations were performed using the CARPentry software package, which is now succeeded by openCARP (http://www.opencarp.org). In particular, we created two regions with a similar size as the early and late RT regions in the patient (RT surface ratio of 0.5), see Figure 5A. We varied the conductance of the rapid delayed-rectifier potassium current (I_Kr_) in these regions to phenocopy the distinct differences in early-vs.-late repolarization. We varied the I_Kr_ conductance of the first region to obtain “normal”, “early” or “very early” repolarization times in that region (by using 100%, 400% and 700% of the original conductance, respectively). In the rest of the heart, we varied the I_Kr_ conductance to obtain “normal”, “late” or “very late” RTs (by using 100%, 25% and 1% of the original conductance, respectively). In all nine combinations of conductances for these regions we subsequently performed an S1-S2 stimulation protocol, where S1 represented sinus-rhythm activation (stimulating multiple locations to get rapid ventricular activation) and S2 was provided at a single location to represent a premature beat, as described previously, to test the inducibility of reentry (1). We varied the S1-S2 coupling interval with 10 ms increments to study the trigger-substrate interaction leading to arrhythmias, and obtain the resulting “vulnerable window” (i.e., the range of S1-S2 coupling intervals resulting in arrhythmias).

These results demonstrate that simulations with larger RT differences (with consequently steeper RT gradients and a larger histogram separation between the early-RT peak and late-RT peak) are more prone to extrasystole-induced arrhythmias. In a simulation with one early RT region and one normal RT region, an early extrasystole (S2) did not block against the RT gradient and thus no arrhythmia was observed (simulation b1 of Figure 5B). In a heart with a very early RT region and a very late RT region, the extrasystole blocked against the RT gradient, traveled around it, and reentered the previously refractory tissue to initiate a reentrant arrhythmia (simulation b2 of Figure 5B). In general, the S1-S2 coupling intervals that resulted in an arrhythmia (the vulnerable time window, red zones in Figure 5C) increased with larger RT differences, in particular when very early RT regions were involved.

Although a direct comparison between the ECGI observations in the patient and simulation results is not possible, an inspection of the patient's RT histogram (Figure 4C, 80 ms separation of the histogram peaks) and the simulated RT histograms (Figure 5C) would suggest the patient's heart may express behavior similar to the “early, normal” simulations (80 ms separation of the histogram peaks) and close to the “early, late” simulations (middle row of Figure 5C, 100 ms separation of the histogram peaks). In the latter simulation, a short-coupled extrasystole (with 300 ms S1-S2 interval) resulted in a sustained arrhythmia. The patient typically showed overall shorter RTs than those simulated, which may be reproduced by changing the basal heart rate in the simulations or using different parameter combinations for the two distinct regions. Although it is challenging to use this modeling approach to exactly replicate the observed RTs, these results show that a clear separation of the two distinct RT regions increases the susceptibility for arrhythmias induced by short-coupled extrasystoles from the early-RT region, with an increasing size of the vulnerable window for hearts with larger RT separation (and thus steeper RT gradients).

## 6. Discussion

Here, we illustrate our novel mechanistic framework “Circle of Reentry,” by characterizing substrate and trigger properties in a patient with “unexplained” SCA. The original three elements of the Triangle of Coumel (trigger, substrate, modulators) are present as the “4 + 1” elements of the Circle of Reentry: both trigger and substrate are subdivided in their spatial and temporal components, and modulating factors are placed outside the circle as they may affect all of these elements (Figure 1C).

Our concept also extends the classical reasoning by Mines about wave length (and wave length adaptation) (16, 17), which states that the length of an activation wave needs to be shorter than the reentrant path length for a reentrant arrhythmia to continue. The Circle of Reentry does not require a predefined path length (which may not explicitly exist at the moment of first unidirectional block), and also remains true for the interaction between multiple wave fronts (which do not have clearly defined wave lengths nor path lengths). The latter is particularly relevant for the interaction between a premature beat (from a single ventricular origin) and the recovery of the preceding sinus beat. Such complex interactions may not be captured by simplified frameworks such as Miles' wave length theory or Coumel's triangle, but could be characterized with detailed investigations and studied with personalized computer simulations that include all elements of the Circle of Reentry, i.e., a Digital Twin solution (18).

In the case of idiopathic VF presented in this paper, ECGI showed signs of abnormal local early repolarization and steep repolarization gradients. Reconstructed epicardial electrograms did not show abnormal conduction (e.g., no fractionation, ST-segment elevation), nor did invasive endocardial voltage mapping. Tissue simulations illustrated how the steep local repolarization gradient could function as line of conduction block for short-coupled beats, facilitating functional reentry. In particular, for large differences between the early and late repolarizing region, the vulnerable window was large. The substrate present in this patient was captured with ECGI only after the sudden cardiac arrest, and it is likely that both the substrate and the coupling interval of the short-coupled beats vary over time in response to dynamic modulators of cardiac electrophysiology, potentially tipping the balance (even steeper RT gradients, even shorter premature beat coupling interval) that resulted in the occurrence of VT and VF in this patient. Genetic analysis did not identify a clear cause for the RT substrate, but did find a genetic variant (of unknown significance) in *TRPM4*, a channel that has been suggested to contribute to ectopic activity (19). Previous studies in idiopathic VF suggest an important role for Purkinje/fascicular system as triggers, and Purkinje potentials preceding premature beats suggest a similar role in our case (20, 21). Comparing the ECG-based allocation of the PVC origin (moderator band) with the first epicardial breakthrough on ECGI (RVOT region) suggests a spread of activation from earliest origin at the moderator band to epicardial breakthrough in a region of early repolarization, potentially through the conduction system and in line with these previous findings.

This patient was treated with quinidine, a class-IA antiarrhythmic agent, which successfully abolished the PVCs (pre-quinidine: 17,675/24 h, post-quinidine: 1/24 h). Repeat-ECGI showed no change in recovery isochrones after quinidine (see Supplementary Figure S1), suggesting that the drug only affected the trigger, not the substrate. By removing one critical factor of the Circle of Reentry, it may prevent new occurrences of VT and VF, although a larger safety margin might be obtained if therapeutic strategies could be identified that could reduce the RT substrate as well. As quinidine is a multichannel blocker, higher doses may have an impact on substrate characteristics as well [and may explain why quinidine is relatively successful in idiopathic VF patients (22)]. In addition to quinidine therapy the patient received an implantable cardioverter defibrillator. She was event-free at 8 years follow-up.

The Circle of Reentry concept could potentially be used to create a clinical risk stratification score, for example by scoring the severity of each of the elements (e.g., burden of PVCs, or steepness of RT gradients) while considering that *all* elements are required for reentry initiation. Such a score could, after prospective clinical validation, potentially guide therapy (e.g., antiarrhythmic drug, trigger ablation or ICD implantation).

Furthermore, the concept of the Circle of Reentry can be generalized to other conditions. For example, we illustrated VF initiation based on a single ventricular beat on top of preceding sinus rhythm RT heterogeneity, but the concept may be extended to arrhythmias that start with multiple focal beats by considering the interaction of a focal beat with the RT of the previous focal beat. Under certain conditions, such focal beats may invert existing RT gradients, generating a new RT substrate and explaining the transition from a focal mechanism to reentry (23).

Additionally, in this patient the heterogeneity of RT stemmed from heterogeneity in repolarization duration. However, RT heterogeneity may also result from heterogeneity in activation, for example due to zig-zag conduction through small conducting channels within the permanently inexcitable scar in post-infarct VTs (24). Moreover, during ischemia, excitability of tissue is not determined by local RT but more directly by post-repolarization refractoriness (25). Thus, the Circle of Reentry may be more broadly applicable if we generalize to local “excitability heterogeneity,” irrespective of whether this is caused by heterogeneity in local activation, repolarization duration, post-repolarization refractoriness, or inexcitable scar; and irrespective of the location of dispersion (LV-vs.-RV, base-to-apex, more localized, or even transmural). This framework may also apply to atrial fibrillation (AF), where the role of dispersion of excitability also appears a key factor (26) and the role of triggers (in particular from the pulmonary veins) is well-known (4). In AF, the excitability dispersion may be caused by a complex interaction between structural abnormalities (fibrosis and fatty infiltrations) and electrical abnormalities (electrical remodeling and adipokines) (27). A clinical risk score, as proposed above for ventricular arrhythmias, may be more challenging for AF (or transmural abnormalities) as the dispersion of excitability may occur at much smaller scales and thus be challenging to detect with imaging, ECG or ECGI.

Similarly, the Circle of Reentry allows to reason about the effect of channelopathies or structural disease, as long as their impact on the four elements is understood. For example, scar in post-infarct hearts has two effects on excitability: there is *no* excitability in scar core, and a large excitability dispersion between the entrance and exit of channels of living tissue within scar, due to (apparent) slowed conduction. On the other hand, some channelopathies have a clear impact on trigger formation. Importantly, our concept suggests that it is the combination of such abnormalities that is critical to the occurrence of reentry. In some cases, there may be a direct, causative relationship between substrate and triggers. For example, under certain conditions extreme RT heterogeneity may act both as a substrate and a cause for triggers: Liu et al. have shown that steep RT gradients may generate premature activations, which then interact with the RT gradients to result in polymorphic VT (“R-from-T”) (28).

Our concept may even apply to arrhythmias of other electromechanical organs. For example, the role of (premature) activation and conduction block has been recently studied in dysrhythmias of the gastrointestinal system (29) and uterus (30), but the role of electrical refractoriness gradients in such organs is still unknown.

Taken together, our study proposes a “Circle of Reentry”, which describes the four key characteristics that are all necessary for reentry initiation (Figure 1C):

(1) The critical timing of the inducing trigger relative to the dispersion of excitability (late enough to excite recovered tissue, early enough to still encounter refractory tissue);
(2) The macroscopic origin of the inducing trigger (coming from a region of early excitability, as to still encounter refractory tissue);
(3) The presence of a steep excitability time gradient (that separates the excitable from refractory tissue and creates a time-consuming deviation for the trigger's activation wave front);
(4) A critical balance between the relative sizes of the tissue with early and late excitability (the early-excitable region should be big enough to allow the trigger to travel for a sufficient time to allow the late-excitable region to recover; and the late-excitable region should be big enough to then take sufficient time for the wave front to travel to allow the early-excitable region to be excitable *again*).

Future research is needed to find the quantitative thresholds that may be applied to each of these characteristics to identify hearts and patients at risk; some initial values for idiopathic VF have been presented in (1). As with Coumel's Triangle of Arrhythmogenesis, modulating factors remain critical and may affect any of the four elements mentioned above.

## 7. Conclusion

Four critical spatiotemporal characteristics of trigger and substrate (and their modulators) are essential to the initiation of reentry, and form the “Circle of Reentry”. Combining this mechanistic reasoning framework with translational investigation of patient cases may help to obtain personalized insights in sudden cardiac arrest and may guide therapy. This concept may also be generalized to other arrhythmias that develop due to excitability dispersion, and its individual components may inform risk stratification and form targets for therapy.

## Data Availability

The original contributions presented in the study are included in the article/**Supplementary Materials**, further inquiries can be directed to m.cluitmans@maastrichtuniversity.nl.
