# Supplementary material for: The circle of reentry: Characteristics of trigger-substrate interaction leading to sudden cardiac arrest
Source: Front Cardiovasc Med. 2023 Apr 17;10:1121517. doi: 10.3389/fcvm.2023.1121517 (PMC10150924; doi:10.3389/fcvm.2023.1121517)
Supplement: Supplementary file 1 [file Datasheet1.docx]

Supplementary Material

The circle of reentry: characteristics of trigger-substrate interaction leading to sudden cardiac arrest

Matthijs JM Cluitmans^*^, Jason Bayer, Laura R Bear, Rachel MA ter Bekke, Jordi Heijman, Ruben Coronel, Paul GA Volders

*** Correspondence:** Matthijs Cluitmans, [m.cluitmans@maastrichtuniversity.nl](mailto:m.cluitmans@maastrichtuniversity.nl)


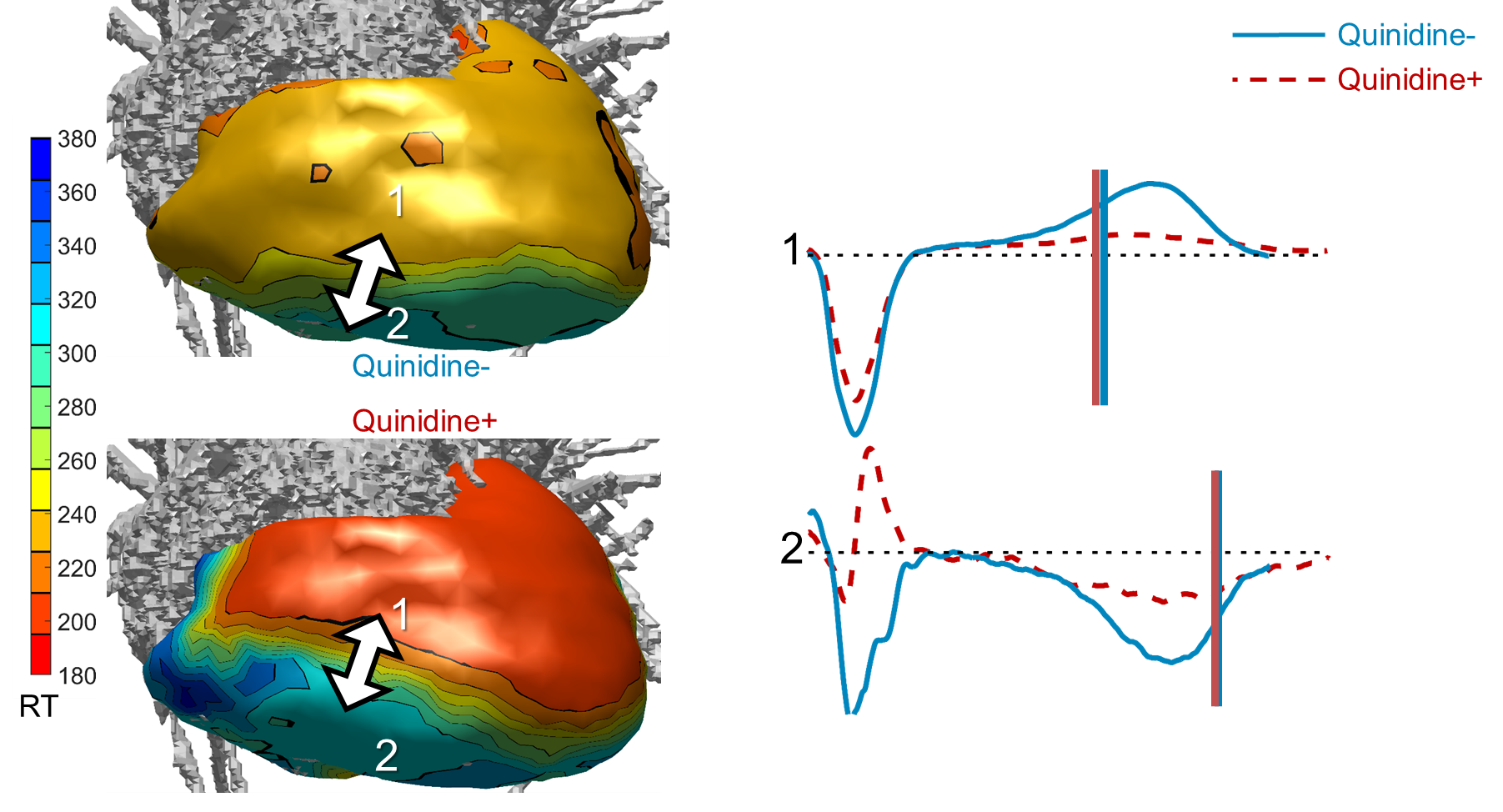


**Supplementary Figure 1.** Electrocardiographic imaging (ECGI) repolarization time (RT) isochrones in the patient case from the main manuscript, before (quinidine-) and during (quinidine+) treatment. There are subtle repolarization differences after quinidine treatment. The global sequence of repolarization (determined by RT defined as the moment of steepest upslope in the local unipolar electrogram) does not change substantially. The RT gradient (highlighted by the arrow) is present both before and during quinidine treatment. Representative local unipolar electrograms on location 1 and 2 are displayed on the right and show that the moments of steepest upslope of the unipolar repolarization wave (representative for local RT, vertical lines) are not different during drug treatment.
